# Multi-Objective Optimization Algorithm to Discover Condition-Specific Modules in Multiple Networks

**DOI:** 10.3390/molecules22122228

**Published:** 2017-12-14

**Authors:** Xiaoke Ma, Penggang Sun, Jianbang Zhao

**Affiliations:** 1 School of Computer Science and Technology, Xidian University, Xi’an 710071, China; psun@mail.xidian.edu.cn; 2 College of Information Engineering, Northwest Agriculture and Forestry University, Yangling, Xianyang 712100, China; zhaojianbang@nwsuaf.edu.cn

**Keywords:** multiple networks, specific modules, multi-objective optimization, network analysis

## Abstract

The advances in biological technologies make it possible to generate data for multiple conditions simultaneously. Discovering the condition-specific modules in multiple networks has great merit in understanding the underlying molecular mechanisms of cells. The available algorithms transform the multiple networks into a single objective optimization problem, which is criticized for its low accuracy. To address this issue, a multi-objective genetic algorithm for condition-specific modules in multiple networks (MOGA-CSM) is developed to discover the condition-specific modules. By using the artificial networks, we demonstrate that the MOGA-CSM outperforms state-of-the-art methods in terms of accuracy. Furthermore, MOGA-CSM discovers stage-specific modules in breast cancer networks based on The Cancer Genome Atlas (TCGA) data, and these modules serve as biomarkers to predict stages of breast cancer. The proposed model and algorithm provide an effective way to analyze multiple networks.

## 1. Introduction

Recent advances in high-throughput biological technologies enable the generation of genome-wide profiles of many patients with various conditions, such as clinical stages, cancer subtypes and time points. Additionally, the network has been proven to be powerful for describing and analyzing the profile data, for which each vertex represents a gene and each edge corresponds to an interaction between a pair of genes. For example, in gene co-expression networks [1], the weight on an edge quantifies the correlation between a pair of genes on the basis of the gene expression profiles. There are various biological networks, such as gene regulation networks [2], signal transduction networks [3], protein–protein interaction (PPI) networks [4], disease networks [5] and gene regulation networks [6,7,8].

The accumulated biological networks provide an opportunity to explore the mechanisms of cells via mining the graph patterns. Great efforts have been devoted to network analysis, for which the graph patterns shed light on the structure–function relations in biology. For example, Taylor et al. [9] analyzed the PPI network and demonstrated that the genes with large degrees (hub genes) play a critical role in the prognosis of breast cancer. Among these graph patterns, module detection in networks has been extensively studied because this plays an important role in revealing the mechanisms of cells. For example, the dense subgraphs in protein interaction networks are very likely to be protein complexes, which are a cornerstone of many biological processes, and together they form various types of molecular machinery that perform a vast array of biological functions [10]. Furthermore, Ideker et al. [11] showed that the pathways through which genes are differentially expressed between two cohorts of cancer patients serve as biomarkers for predicting cancer metastasis.

Thus, great efforts have been devoted to discovering modules in networks [12,13,14,15,16]. These algorithms mainly differ in their characterization of the module structure and their strategy of module discovery. Although these methods are promising in discovering modules in networks, they solely focus on identifying a module in a network. In fact, each gene has multiple attributes, indicating that an interaction cannot fully characterize the relation between a pair of genes. For example, proteins possess multiple features, such as physical and co-localization features [17]. To this end, the interactome of proteins for some organisms is up to several distinct network layers accounting for different genetic and physical interactions, each layer containing thousands of protein–protein relationships [18]. The cancer deleterious is dynamic, implying that multiple networks are required to model the progression of diseases, for which each network corresponds to a specific stage.

Fortunately, many algorithms have been developed to extract modules in multiple networks [14,15,16,17,18,19,20,21,22]. For instance, Ma et al. [20,21] designed the *M-Module* algorithm to discover common modules within multiple networks, which can trace the dynamics of pathways associated with cancer progression. Kelly et al. [16] extracted the conserved modules in multiple networks for various species, which can infer homologous proteins across species. These results demonstrate that discovering graph patterns within multiple networks is promising.

Although great efforts have been devoted to common module detection, few attempts have been made to extract the condition-specific modules in multiple networks, because it is difficult to characterize the specific modules. To accurately depict the specific modules, we must balance the specificity and modularity of modules. Currently, the available algorithms handle this issue by separating the specificity and modularity. Specifically, for each condition, a specific network is constructed for which the edge weight quantifies the specificity of the corresponding edge across all the conditions (details are presented in the next section). Then, module search algorithms for the constructed network, such as WGCNA [23], are employed to obtain the modules. The advantage of this strategy is simplicity, as any module search algorithm can be directly applied. However, it is difficult to achieve a good trade-off between the specificity and modularity because these are independent; this is the major motivation of the present study.

To overcome this problem, an efficient heuristic algorithm is proposed for the specific modules in multiple networks (SMMN), which discovers the condition-specific modules by considering multiple networks without collapsing networks [24]. However, the SMMN algorithm transforms the problem into a single objective optimization, which cannot fully characterize the condition-specific modules in multiple networks. However, it has been shown that intelligent algorithms, such as genetic algorithms (GAs) and particle swarm optimization (PSO), provide an effective strategy to address the optimization problems. For example, Kowk et al. showed that PSO algorithms are effective and efficient in image processing [25,26], industry applications [27] and graph clustering [28]. Knowles et al. [29] demonstrated that the multiobjective optimization is promising in bioinformatics. Inspired by the intelligent algorithms, we present a multi-objective genetic algorithm for condition-specific modules (MOGA-CSM) for condition-specific modules in multiple networks. We demonstrate that the MOGA-CSM outperforms state-of-the-art methods by using artificial and real-world multiple networks.

The rest of the paper is organized as follows: Section 2 proposes the mathematical model and algorithm. The related materials are presented in Section 3. The experimental results are provided in Section 4. The conclusion is discussed in Section 5.

## 2. Methods

**Figure 1:**
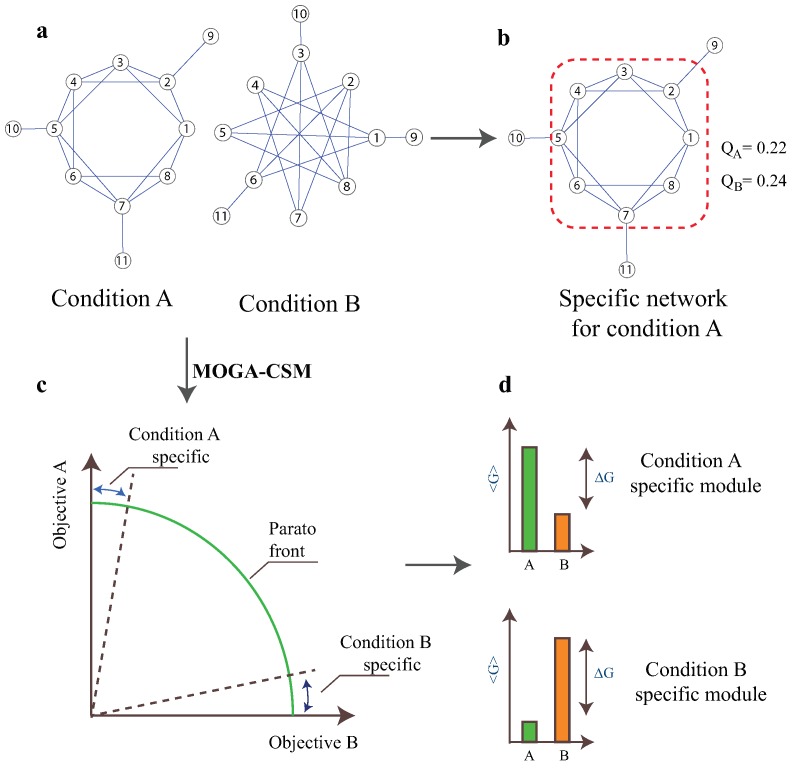
A schematic example of the limitations of the available approaches for condition-specific modules and the overview of the multi-objective genetic algorithm for condition-specific modules (MOGA-CSM). (**a**,**b**) Limitation of the current algorithms: (**a**) Two networks under conditions A and B; (**b**) The condition A-specific networks, for which the module is surrounded by the red dashed line, are specifically obtained by the WGCNA algorithm. The connectivity of the condition A-specific module obtained by the current algorithm in network B is even stronger than that in network A, which contradicts intuition; (**c**,**d**) Overview of the proposed algorithm: (**c**) the MOGA-CSM transforms the condition-specific module detection into a multi-objective optimization problem, for which the specific modules can be obtained by maximizing the connectivity of modules and minimizing the connectivity of modules in other networks; and (**d**) the connectivity of specific modules obtained by MOGA-CSM.

In this section, we first discuss the mathematical model for the condition-specific modules and then describe the MOGA-CSM. It is shown that the traditional algorithms cannot effectively characterize the specificity of modules within multiple networks [24] (Figure 1a,b). The ultimate goal is to develop a multi-objective GA for this issue (Figure 1c,d).

### 2.1. Multi-Objective Mathematical Model

Let {1,2,…,M} be a finite set of conditions, and let the attached subscript *m* be the value of the variable under condition *m*. The multiple network G is defined as a sequence of networks $G=\{G_{1},G_{2},\ldots ,G_{M}\}$, where $G_{m}$ is the network at condition *m* with a vertex set *V* and an edge set $E_{m}$. The adjacency matrix for G is defined as $W={\left(w_{ijm}\right)}_{n\times n\times M}$, where *n* is the number of genes in G (i.e., n=|V|) and $w_{ijm}$ is the weight on the edge connecting the *i*th and *j*th gene in $G_{m}$.

Given network $G_{m}=\left(V,E_{m}\right)$, the module detection aims at obtaining a hard partitioning of *V*, that is, $\left\{C_{1m},C_{2m},\ldots ,C_{km}\right\}$ (denoted by ${\left\{C_{im}\right\}}_{i=1}^{k}$, such that $C_{im}\cap C_{jm}=\emptyset$ if i≠j and $V=\sum_{i}C_{im}$), where *k* is the number of modules. Given the partitioning ${\left\{C_{im}\right\}}_{i=1}^{k}$ of $G_{m}$, an n×k index matrix *X* is constructed to represent the memberships of genes such that columns correspond to modules and rows correspond to genes. Element $x_{ij}$ = 1 if the *i*th gene belongs to module $C_{jm}$ and is 0 otherwise. The connectivity of module $C_{tm}$ in network $G_{\iota }$ is quantified by the modularity *Q* [30]. According to [24], the overall function of the condition-specific modules for the condition *m* is defined as

(1) $$\begin{array}{cc} \max_{X} & F(X)\\ & x_{ij}\in \left\{0,1\right\}\\ s.t. & \sum_{j=1}^{k}x_{ij}=1\\ & \sum_{i=1}^{n}x_{ij}\geq 1 \end{array}$$

where $F\left(X\right)=(F_{1}\left(X\right),\ldots ,F_{m}\left(X\right),\ldots ,F_{M}\left(X\right))$ are the multi-objective functions, for which $F_{i}\left(X\right)=Q_{i}\left({\left\{C_{tm}\right\}}_{t=1}^{k}\right)$ for i≠m, and $F_{m}\left(X\right)=1-Q_{i}\left({\left\{C_{tm}\right\}}_{t=1}^{k}\right)$. Because this is an NP-hard problem, we employ a heuristic algorithm to obtain the solution for Equation (1).

Differently from [24] using a single objective optimization problem, we present a GA to directly address the multi-objective optimization problem in Equation (1). We first introduce the *Pareto front* for solutions. Given two solutions $X^{\left[1\right]}$ and $X^{\left[2\right]}$ to the multi-objective optimization problem in Equation (1), $X^{\left[1\right]}$ is dominated by $X^{\left[2\right]}$, denoted by $X^{\left[1\right]}≺X^{\left[2\right]}$, if and only if

$$\forall i:F_{i}\left(X^{\left[1\right]}\right)\leq F_{i}\left(X^{\left[2\right]}\right)\wedge \exists i,s.t.F_{i}\left(X^{\left[1\right]}\right)<F_{i}\left(X^{\left[2\right]}\right)$$

Instead, a nondominated solution is one for which an improvement in one objective requires a degradation of the other(s). The set of these nondominated solutions is called the *Pareto front*.

### 2.2. The MOGA-CSM

GAs are a class of adaptive search methods inspired by natural evolution [31], which evolves a population of individuals using the operators of *crossover* and *mutation*. Each individual represents a candidate solution to the problem in Equation (1). The *fitness value* of an individual quantifies how good it is with respect to the other solutions in the population. The crossover operator generates an individual by combining two individuals in the population, while the mutation operator randomly alters the individual. GAs, for example, the *nondominated sorting genetic algorithm (NSGA-II)*, have been successfully applied to multi-objective optimization problems (MOGA) [32]. Recently, Gu et al. [33] proposed an innovative semi-active storey isolation system by utilizing the NSGA-II based on the dynamic crowding distance (DCD), which significantly improved the performance. GAs have been widely applied to network clustering [34].

**Figure 2:**
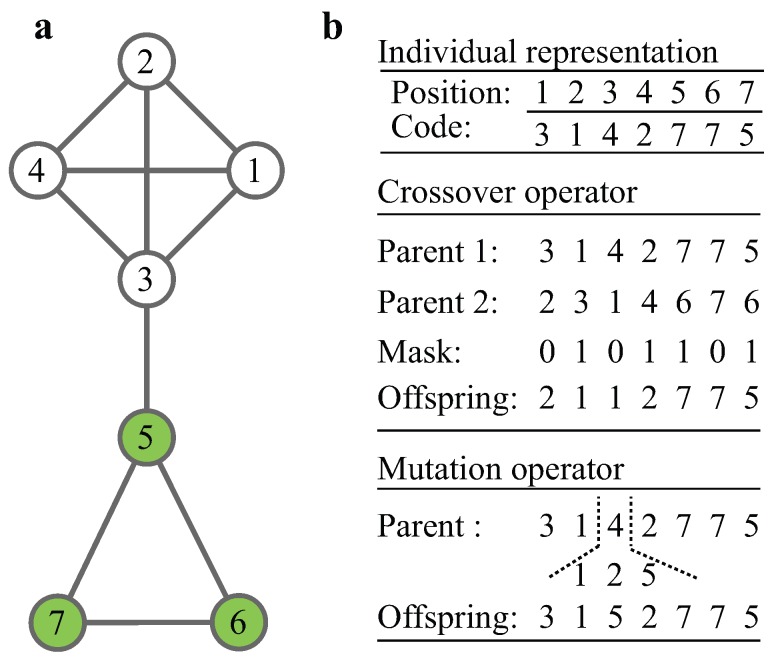
Illustration of procedure of the multi-objective genetic algorithm for condition-specific modules (MOGA-CSM): (**a**) A network with seven vertices partitioned into two modules, {1,2,3,4} and {5,6,7}; (**b**) Procedure of MOGA-CSM: top panel corresponds to a locus-based representation of the network on the left; middle panel contains the example of uniform crossover; bottom panel represents the mutation.

**Individual representation:** The locus-based adjacency representation is adopted [35,36,37]. In this graph-based representation, an individual is denoted by $P=(g_{1},\ldots ,g_{n})$, where $g_{i}$ is one of the neighbors of node *i* such that $\left(i,g_{i}\right)$ is an edge belonging to one of the modules of the graph. The schematic example of representation for a graph (Figure 2a) is illustrated in Figure 2b, where the two modules are encoded.

To decode the module within an individual, the disjoint set algorithm [38] is employed, in which the modules correspond to a set of disjoint dynamic sets, where each set is represented by a rooted tree. The root is defined as the representative, and the rest node *i* of the tree points only to its parent parent(i). The level of node *i* is defined as the length of the shortest path connecting *i* to the root, denoted by level(i). At the beginning, the decode procedure initializes each vertex as a set (step 1); that is, the parent of *i* is itself and the level is 0. Then, for each edge $\left(i,g_{i}\right)$, it tracks the roots of the tree(s) of *i* and $g_{i}$, denoted by $r_{1}$ and $r_{2}$ (step 2). If the node *i* and $g_{i}$ belong to various trees, that is, $r_{1}\leq r_{2}$, it merges the two trees as a new tree (step 3). Otherwise, it updates the levels of genes within the tree (step 4).

**Algorithm 1** Decoding Procedure**Input:**  *P*: an individual of the population. **Output:**  $X_{P}$: the module structure of *P*. 1:  For each node i∈V, set parent(i)=i and level(i)=0. 2:  For each edge $\left(i,g_{i}\right)$, find the roots of *i* and $g_{i}$, denoted by $r_{1}$ and $r_{2}$, respectively. 3:  If $r_{1}=r_{2}$, update $level\left(r_{2}\right)=level\left(r_{1}\right)+1$; else goto step 4. 4:  If $level\left(r_{1}\right)>level\left(r_{2}\right)$, update $parent\left(r_{2}\right)=r_{1}$; else update $parent\left(r_{1}\right)=r_{2}$. 5:  **return** Disjoint sets.

**Crossover operator:** Given two parents in population $P^{\left[1\right]}=\left(g_{1}^{\left[1\right]},\ldots ,g_{n}^{\left[1\right]}\right)$ and $P^{\left[2\right]}=\left(g_{1}^{\left[2\right]},\ldots ,g_{n}^{\left[2\right]}\right)$, the child $S=(g_{1},\ldots ,g_{n})$ is generated by randomly selecting each component from one of the parents; that is, $g_{i}$ is either $g_{i}^{\left[1\right]}$ or $g_{i}^{\left[2\right]}$. This procedure is fulfilled by a random binary mask: when mask is 0, $g_{i}=g_{i}^{\left[1\right]}$; otherwise $g_{i}=g_{i}^{\left[2\right]}$. The advantage of the crossover is to maintain node connections in the child individual.

**Mutation operator:** Given an individual $P^{\left[1\right]}=\left(g_{1},\ldots ,g_{n}\right)$, the mutation operator randomly changes the value of $g_{i}$. To guarantee the connections, only the neighbors of node *i* are candidates for replacing $g_{i}$.

The pseudo-code of MOGA-CSM is presented in Algorithm 2. Given the multiple networks $G=\{G_{1},G_{2},\ldots ,G_{M}\}$ and the condition *m*, MOGA-CSM generates a population of random individuals. Specifically, given an individual $P=(g_{1},\ldots ,g_{i})$, we randomly select one vertex from the neighbors of vertex *i* and assign it $g_{i}$. After the population is generated, it decodes the individuals of the population to produce the partitioning and evaluates the objective values. The individuals are ranked according to the Pareto dominance. The crossover and mutation operators are employed to create the new population. Finally, the solutions in the Pareto front are returned, where each of them corresponds to a trade-off among multiple functions. Therefore, a criterion is required to select one solution with respect to another. We choose the solution with the maximum modularity for $G_{m}$ on the basis of the fact that the Pareto front has already selected the nondominated solutions that best satisfy all the functions.

**Algorithm 2** The MOGA-CSM**Input:**  G: the involved multiple networks.  *m*: the specific condition. **Output:**  ${\left\{C_{tm}\right\}}_{t=1}^{k}$: the condition-specific modules. 1:  Create a population of random individuals for $G_{m}$. 2:  Decode each individual *P* of the population using the decoding procedure. 3:  Obtain the rank of each individual according to nondomination rank. 4:  Generate new offspring using the crossover and mutation operators. 5:  Combine the parents and offspring into a new pool and rank them. 6:  Select the individuals with lower rank for the next generation. 7:  If the termination criterion is not satisfied, goto step 1; otherwise, goto step 8. 8:  **return**
${\left\{C_{tm}\right\}}_{t=1}^{k}$ with the maximum modularity.

### 2.3. Algorithm Analysis

In terms of space complexity, the space for the adjacency matrix of multiple networks is $O(n^{2}M)$. For each network, the space for the population is O(np), where *p* is the size of the population. The space complexity of the indicator matrix for modules is O(nkM), where *k* is the number of modules. Because k≪n, the total space complexity of the proposed algorithm is $O(n^{2}M)$.

In terms of time complexity, the MOGA-CSM makes use of *NSGA-II* to rank the non-dominance [34], which requires time $O(tp{\left(\log p\right)}^{h-1})$, where *t* is the number of generations, *p* is the size of the population, and *M* is the number of objective functions. Because MOGA-CSM optimizes *M* networks, the time complexity is $O(tp{\left(\log p\right)}^{M-1})$. For each generation, the crossover needs O(n) time, and mutation requires O(1) time. Furthermore, the decoding procedure requires O(nlogn) time [38]. Thus, the total time complexity of MOGA-CSM is $O(tp{\left(\log p\right)}^{M-1}n\log n)$.

## 3. Materials

### 3.1. Statistical Significance of Specific Modules

The statistical significance of specific modules was computed on the basis of the null score distribution of specific modules generated using randomized networks. Each network was completely randomized 100 times by degree-preserved edge shuffling. To construct the null distribution for specific module scores, we performed the MOGA-CSM on the randomized networks. Using the null distribution, the empirical *p*-value of a specific module was calculated as the probability of the module having the observed score or greater by chance; *p*-values were corrected for multiple testing using the method of Benjamini–Hochberg [39]. An adjusted *p*-value of 0.05 was considered as significant.

### 3.2. Features for Support Vector Machine on Specific Modules

Given a module *C*, we normalize the expression level of each gene across all samples using the z-score transformation [11], denoted by $Exp_{ij}$ for the *i*th gene and *j*th patient. For each sample *j*, the activity score of the *k*th module is defined as the average gene expression of all genes within the module, that is,

(2) $$e_{C}=\sum_{i\in C}Exp_{ij}/\sqrt{\left|C\right|}$$

where |C| is the number of genes in *C*. For each patient sample, a feature vector is constructed by all modules.

### 3.3. Artificial Networks

The artificial network is introduced in [30]. In each network, 128 nodes are grouped into 4 clusters of equal size. Every node has an average degree of 16 and shares $k_{out}$ edges connecting nodes outside of the module to which it belongs. As parameter $k_{out}$ increases from 1 to 8, the detection of clusters in the networks becomes increasingly difficult.

### 3.4. Breast Cancer Networks

The gene expression data for breast cancer was downloaded from the TCGA Data Portal, where the clinical stage information for patients is also available. The RPKM (Reads Per Kilobase per Million mapped reads) values are used. There are 715 samples across four stages (stage I: 119; stage II: 407; stage III: 189).

For each stage, we construct a gene co-expression network, where the edge weight is defined as the absolute value of the Pearson correlation of the gene expression profiles of a pair of genes. To remove indirect correlation due to a third gene, we use the first-order partial Pearson correlation coefficient (PCIT package [40]). The breast cancer networks contain 6643 genes and about 2.6 million edges.

## 4. Results

To fully test the performance of the proposed algorithm, we compared MOGA-CSM with the available algorithms. We note that the current approaches differ greatly on their strategy of how to extract the modules from the constructed condition-specific networks. Thus, we adopted three well-known algorithms, including the WGCNA [23], spectral clustering (SPEC) [41] and nonnegative matrix factorization (NMF) algorithms [42]. The reason that these algorithms were selected was that they achieve excellent performance in detecting modules in networks.

Two types of networks, both artificial and real biological networks, were employed for a comparison among various algorithms. The artificial networks were adopted to test the accuracy of the MOGA-CSM, and the breast cancer networks were used to determine the the applicability of the proposed algorithm in discovering condition-specific modules in real networks with a strong background. The parameters for the MOGA-CSM were set as follows: crossoverrate=0.8 and mutationrate=0.2. The reason was that in general, a high crossover rate and low mutation rate are suggested in GAs. Furthermore, we set elitereproduction=10% of the population size, and the number of generations as 500 (how the parameters affect the performance is discussed in the following section).

### 4.1. Benchmarking Performance of the Artificial Networks

In the artificial networks, we constructed two networks by combining a network with a known module structure (Materials) and a size-matched random network. Therefore, the modules in the benchmark network were specific modules, because the random network was not expected to exhibit a modular structure. To quantify the performance of the algorithms, the modularity *Q* was used.

**Figure 3:**
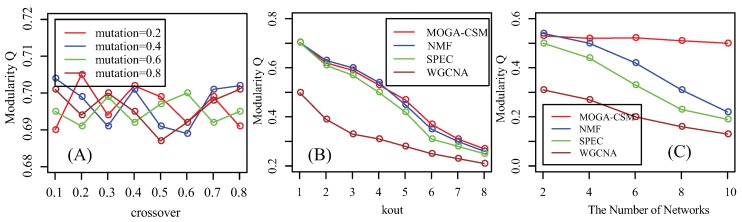
Performance of the compared algorithms on artificial multiple networks. (**A**) Parameter effect: modularity for different combinations of crossover and mutation rates for the artificial networks; (**B**) Performance as a function of the amount of noise in simulated networks, where modularity *Q* is used as the performance measure. Shown here are average *Q* values of 50 runs of each method at each noise level; (**C**) Performance as a function of the number of networks in the artificial networks, where *Q* is used as the performance measure.

Prior to giving the performance of the algorithms, we first investigate the effects of the parameters for MOGA-CSM on the artificial networks. The results are shown in Figure 3A, where the crossover rate ranges from 0.1 to 0.8 with a gap 0.1 and the mutation rate ranges from 0.2 to 0.8 with a gap 0.2. It can be observed that they do not present high variation.

We compare the WGCNA, SPEC, NMF and MOGA-CSM algorithms on the artificial networks in terms of accuracy, as shown in Figure 3B. From this, we conclude that the performance of the algorithms decreases dramatically as $k_{out}$ increases from 1 to 8, because the module structure becomes fuzzy as $k_{out}$ increases. For example, the *Q* value is 0.7 when $k_{out}$ = 1, and it is 0.22 when $k_{out}$ = 8. Furthermore, the proposed algorithm has a similar performance to NMF when $k_{out}\leq 4$, while it outperforms NMF if $k_{out}>4$. Furthermore, both MOGA-CSM and NMF are superior to the SPEC and WGCNA algorithms. The SPEC algorithm is inferior to others, indicating that the spectral features are insufficient to characterize the specific modules.

As shown in Section 2, the available methods are sensitive to the number of networks. Therefore, we investigated whether the proposed algorithm is also sensitive to the number of networks. We increased the number of random networks from 2 to 10 and tested the performance of various algorithms, as shown in Figure 3C. From this, we conclude that the performance of NMF, SPEC, and WGCNA decreases dramatically as the number of networks increases. However, the MOGA-CSM is not sensitive to the number of networks. The results demonstrate that the proposed algorithm is more accurate and robust than state-of-the-art approaches in discovering condition-specific modules.

### 4.2. Benchmarking Performance of the Breast Cancer Networks

**Figure 4:**
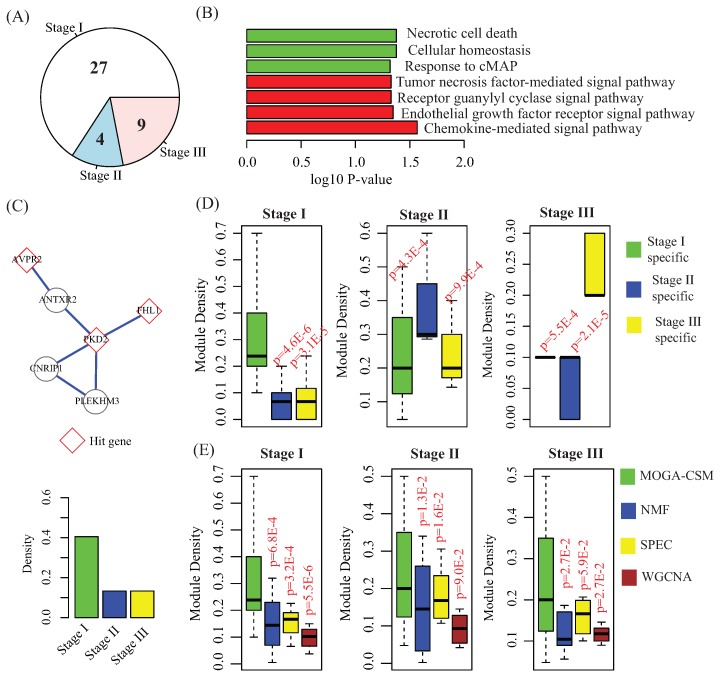
Performance of the compared algorithms on the TCGA breast cancer networks. (**A**) Piechart for the number of stage-specific modules obtained by the multi-objective genetic algorithm for condition-specific modules (MOGA-CSM); (**B**) the barplot for the functions of genes within specific modules, where the red color indicates the genes within stage I-specific modules and green indicates the genes within stage II- and III-specific modules; (**C**) a schematic example of a stage I-specific module obtained by the MOGA-CSM, where the top panel is the topological structure of the module and the bottom panel contains the density of the module in each network; (**D**) distribution of density of stage-specific modules in each network; (**E**) comparison of various algorithms in terms of distribution of density of specific modules obtained by algorithms in each network.

On the basis of the clinical stages for breast cancer, we constructed a gene co-expression network for each stage (Materials). By applying the MOGA-CSM to the breast cancer networks, we obtained 27 (stage I), 5 (stage II) and 9 (stage III) specific modules (Figure 4A).

The homeostasis has been proven to be a critical complex for breast cancer diagnosis and therapy [43]. Remarkably, the MOGA-CSM obtained a stage I-specific module that was significantly enriched by homeostasis (*p*-value = 1.6 × $10^{-2}$, corrected by BH test), as shown in the top panel of Figure 4C. There were six genes (*ANTXR2*, *FHL1*, *AVPR2*, *PLEKHM3*, *PKD2*, and *CNRIP1*), for which genes PKD2, FHL1 and AVPR2 had the function homeostasis. To check whether the module was stage I-specific, we calculated the connectivity of the modules in all three networks, as shown in the bottom panel of Figure 4C. The density of the module in the stage I network was 0.4, while the density in the stage II and III networks was 0.13. These results met our expectation, because the connectivity was strong in the stage I network and weak in others.

Then, we checked the functions of the genes within the stage-specific modules, as shown in Figure 4B. We found that the genes within stage I-specific modules were more likely to be enriched by the signaling pathways (red bars), such as the tumor necrosis factor-mediated signaling pathway (*p*-value = 2.7 × $10^{-2}$, corrected by BH test), the receptor guanylyl cyclase signaling pathway (*p*-value = 4.5 × $10^{-2}$, corrected by BH test), and the endothelial growth factor receptor signaling pathway (*p*-value = 4.7 × $10^{-2}$, corrected by BH test). These signaling pathways are critical for breast cancer [44].

To check the specificity of the modules obtained by MOGA-CSM, we compared the distribution of densities of modules for each network. The results are shown in Figure 4D, where it is indicated that the connectivity of the specific modules can capture the specificity, because the modules are well connected in the corresponding network and weak in others. For example, the density of stage I-specific modules is more significant than that of modules in networks at stage II and III (stage I vs. stage II: *p*-value = 4.6 × $10^{-6}$; stage I vs. stage III: *p*-value = 3.1 × $10^{-5}$, Student’s *t*-test).

Finally, we compared the WGCNA, SPEC, NMF and MOGA-CSM algorithms in terms of discovering the condition-specific modules by applying them to the breast cancer networks. We compared the distribution of densities of the modules obtained for each algorithm, which are shown in Figure 4E. From these, it is easy to conclude that the MOGA-CSM is significantly better than the others, as the density of the modules obtained by MOGA-CSM was much higher than that of others. For instance, in the stage I network, the means of the density of the modules were 0.22 (MOGA-CSM), 0.17 (NMF), 0.19 (SPEC), and 0.12 (WGCNA) (MOGA-CSM vs. NMF: *p*-value = 6.8 × $10^{-4}$; MOGA-CSM vs. SPEC: *p*-value = 3.2 × $10^{-4}$; MOGA-CSM vs. WGCNA: *p*-value = 5.5 × $10^{-6}$, Student’s *t*-test). These results imply that the proposed algorithm is more accurate than state-of-the-art approaches for the specific module detection in biological networks.

### 4.3. Stage-Specific Modules Serve as Biomarkers to Predict Breast Cancer Stages

Taylor et al. [9] showed that the hub genes are predictive for breast cancer diagnosis. Ideker et al. [11] demonstrated that the modules can serve as biomarkers to predict metastasis of breast cancer. Thus, we hypothesized that the stage-specific modules could also be used to predict the stages of breast cancer.

**Figure 5:**
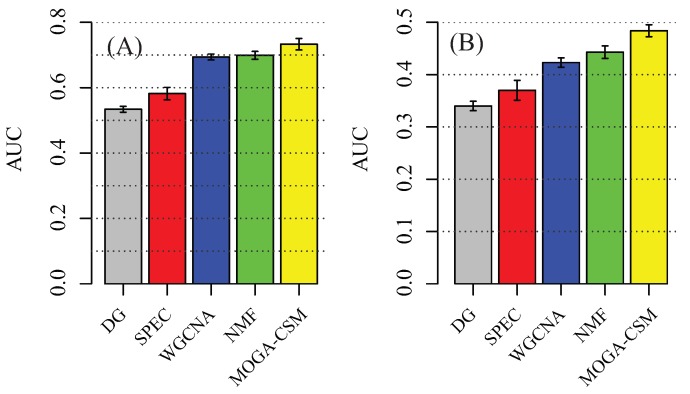
Subtype-specific methylation modules improve the accuracy of breast cancer stage classification using 50 independent five-fold cross-validations. (**A**) Classification accuracy of breast cancer stages using different feature sets, including the stage-specific modules obtained by various algorithms. Accuracy is defined as the number of patient samples correctly classified. The *Y*-axis is the accuracy and the error bar is for the standard deviation; (**B**) External validation by training on TCGA data and testing on external data.

For a baseline comparison, we compared the classification accuracy by using the following feature sets: stage-specific modules generated by SPEC, NMF, WGCNA and MOGA-CSM. We trained the support vector machine (SVM) classifier to perform multi-class classification. The SVM employs the accuracy (percentage of patients that are corrected classified) to measure performance. The results of TCGA breast cancer data by using five-fold cross-validation are presented in Figure 5A. The stage-specific modules obtained by SMMN were more discriminative than the others. Specifically, the MOGA-CSM had a significantly higher accuracy than WGCNA (73.3% vs. 69.9%). The WGCNA algorithm had a similar performance to NMF, and they outperformed the other methods. These results demonstrate that the stage-specific modules obtained by MOGA-CSM capture the specificity of pathways for breast cancer progression.

To further validate the performance of various algorithms, we evaluated the performance of the SVM classifiers by using external data (GSE5874). We trained the SVM classifier on the TCGA data and tested on an external microarray dataset. The consistent results indicated that the performance was not due to the hidden confounding factors in the TCGA dataset (Figure 5A). The accuracy of MOGA-CSM was 48.4%, while the accuracy was 44.3%, 42.3%, 37.1%, and 34.3% for NMF, WGCNA, SPEC and differentially expressed genes (DG), respectively. The results show that the proposed algorithm is better than the available approaches in terms of discovering specific modules in multiple networks.

### 4.4. Benchmarking Performance of Cancer Co-Methylation Networks

To fully explore the performance of the proposed algorithms, we compared these algorithms by using the cancer subtype long non-coding RNA co-methylation networks [45]. There are four subtypes of breast cancer, Luminal A, Luminal B, Her2 and Basal-like. For each subtype, there is a corresponding long non-coding RNA gene co-methylation network.

**Figure 6:**
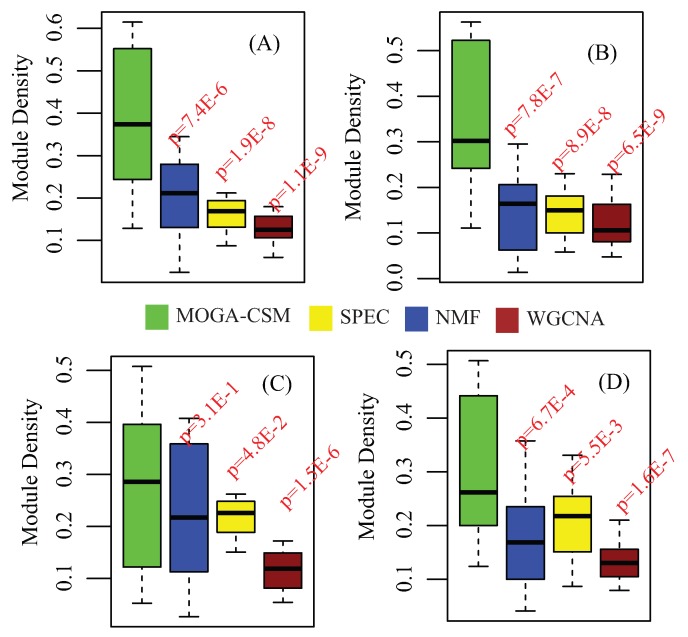
Performance of the compared algorithms on the multiple subtype co-methylation networks for long noncoding RNA networks. Distribution of density of subtype-specific modules in each subtype: (**A**) Luminal A; (**B**) Luminal B; (**C**) Her2 and (**D**) Basal-like.

The results are shown in Figure 6, where the distributions of the graph density of the obtained subtype-specific modules are presented. From these panels, it is easy to assert that the proposed method significantly outperforms the others, because the modules are more specific in terms of density for almost all the subtypes. Therefore, we assert that the proposed method is promising in discovering the condition-specific modules.

## 5. Discussion and Conclusions

Recent technology has enabled the possibility of generating multiple genomic profiling of biological samples for different stages or time points. However, the systematic integrative analysis of multiple-stage (or time-point) data associated with disease progression or cell differentiation for discovering biological relevant patterns is currently lacking. The accumulated multiple networks provide an opportunity to explore the underlying mechanisms of diseases. Although great efforts have been devoted to multiple networks analysis, few attempts have been made to extract the specific modules in multiple networks.

The available algorithms first construct a specific network by compressing the multiple networks. Then, they discover modules in the constructed networks. The strategy is criticized for its low accuracy, because of the separation of specificity and modularity. To overcome this problem, we characterize the specific modules on the basis of the topology of multiple networks rather than the constructed network, which provides a better characterization of modules. Then, a multi-objective optimization model is developed for specific module detection in multiple networks. Finally, a multi-objective optimization algorithm is designed to obtain specific modules. The results demonstrate that the proposed algorithm is better than the current approaches. We wish to point out several unique insights: (i) the integrative analysis of multiple networks without collapsing them is promising, which is overlooked by the available methods; and (ii) the biologically inspired computational approaches, such as GAs, provide an efficient tool to extract the graph patterns in multiple networks.

For further research, we see ample opportunities to improve on the basic concept of the MOGA-CSM. First, although this study uses breast cancer as a proof-of-principle, it is flexible to any diseases, as it is a generalized framework for any cohort of patients with various conditions. Second, data integration might further expand the applicability of the proposed model and algorithm.
